# 2836. Prostate Abscess due to *Staphylococcus aureus*: a Retrospective Case Series

**DOI:** 10.1093/ofid/ofad500.2446

**Published:** 2023-11-27

**Authors:** Zachary Pellis, Steven Mudroch, Stephanie Spivack

**Affiliations:** University of Chicago Medical Center, Philadelphia, Pennsylvania; Temple University Health System, Philadelphia, Pennsylvania; Temple University Health System, Philadelphia, Pennsylvania

## Abstract

**Background:**

Most cases of prostate abscess are due to gram-negative enteric bacteria, but there are increasing reports of *Staphylococcus aureus* as a causative pathogen. Due to being an uncommon diagnosis, there are no current guidelines for the evaluation and treatment of *S. aureus* prostate abscesses. We examined the epidemiology and management of prostatic abscess due to *S. aureus* at an academic, urban tertiary center.

**Methods:**

In this retrospective analysis, we identified patients with diagnosis of prostate abscess and concomitant culture with *S. aureus* who were aged 18 years or older and hospitalized at Temple University Hospital between 2016 and 2022. Cases were identified if they had both (1) a diagnosis code consistent with prostate abscess; and (2) positive blood, urine, or abscess cultures with *S. aureus*. Each case was reviewed to verify clinical or imaging criteria for diagnosis. Data regarding risk factors, interventions, and outcomes were compiled in REDCap, a de-identified electronic database for data management.

**Results:**

We identified 18 patients who met inclusion criteria, with median [IQR] age of 50 years [40-70]. Computed tomography was used in all cases for identifying an abscess. The most common risk factors were intravenous drug use (IVDU) 55.6% [10/18] and diabetes 50% [9/18]. Culture positivity was 100% [6/6] for abscess fluid, 61.1% [11/18] for urine, and 56.3% [9/16] for blood. Of these, methicillin resistance was detected in 72.2% [13/18] of cases. Computed tomography was used in all cases for identifying an abscess. 61.1% [11/18] of patients underwent procedural interventions for treatment. Infectious Diseases consultation resulted in a change in management in 92.8% [13/14] of cases, with a median length of antibiotic administration of 42 days. 66.7% [6/9] of the bacteremic patients had additional infectious complications such as endocarditis or osteomyelitis.

**Conclusion:**

Prostate abscess due to *S. aureus* should be considered in patients presenting with lower urinary tract symptoms and a history of injection drug use or diabetes. Evaluation should include imaging and cultures, particularly from blood. Treatment involves prolonged antibiotics and urology consultation for consideration of procedural intervention.

**Disclosures:**

**All Authors**: No reported disclosures

